# BHK-21 Cell Clones Differ in Chikungunya Virus Infection and MXRA8 Receptor Expression

**DOI:** 10.3390/v13060949

**Published:** 2021-05-21

**Authors:** Peiqi Yin, Margaret Kielian

**Affiliations:** Department of Cell Biology, Albert Einstein College of Medicine, New York, NY 10461, USA; peiqi.yin@einsteinmed.org

**Keywords:** alphavirus, chikungunya virus, receptor, MXRA8, BHK-21 cells

## Abstract

Baby hamster kidney-21 (BHK-21) cells are widely used to propagate and study many animal viruses using infection and transfection techniques. Among various BHK-21 cell clones, the fibroblast-like BHK-21/C-13 line and the epithelial-like BHK-21/WI-2 line are commonly used cell clones for alphavirus research. Here we report that BHK-21/WI-2 cells were significantly less susceptible to primary infection by the alphavirus chikungunya virus (CHIKV) than were BHK-21/C-13 cells. The electroporation efficiency of alphavirus RNA into BHK-21/WI-2 was also lower than that of BHK-21/C-13. The growth of CHIKV was decreased in BHK-21/WI-2 compared to BHK-21/C-13, while primary infection and growth of the alphavirus Sindbis virus (SINV) were equivalent in the two cell lines. Our results suggested that CHIKV entry could be compromised in BHK-21/WI-2. Indeed, we found that the mRNA level of the CHIKV receptor MXRA8 in BHK-21/WI-2 cells was much lower than that in BHK-21/C-13 cells, and exogenous expression of either human MXRA8 or hamster MXRA8 rescued CHIKV infection. Our results affirm the importance of the MXRA8 receptor for CHIKV infection, and document differences in its expression in two clonal cell lines derived from the original BHK-21 cell cultures. Our results also indicate that CHIKV propagation and entry studies in BHK-21 cells will be significantly more efficient in BHK-21/C-13 than in BHK-21/WI-2 cells.

## 1. Introduction

Chikungunya virus (CHIKV) is an enveloped positive-strand RNA virus that belongs to the *Alphavirus* genus in the *Togaviridae* family [1,2,3]. CHIKV is an arbovirus that is transmitted by mosquito vectors. CHIKV infection of humans causes chikungunya fever, which is associated with acute and chronic symmetrical polyarthralgia. Chronic arthritis and joint pain can persist for months to years after the initial infection [3]. Since its discovery in Tanzania in 1952, CHIKV has been known to be endemic to parts of Africa [4]. In 2004, CHIKV reemerged to cause an outbreak that expanded to countries around the Indian Ocean, with an estimated 6 million cases [3,5,6,7]. During these outbreaks new CHIKV variants arose that were more efficiently transmitted by mosquito vectors [3,8]. CHIKV was first reported in the Western Hemisphere in 2013 and has rapidly spread across the Americas to cause more than a million cases [9,10]. No approved vaccine or antiviral therapy for CHIKV is currently available.

Alphavirus genomes encode the four non-structural replicase proteins, and the major structural proteins capsid, E2 and E1 (reviewed in [2,11]). Capsid packages the genome into the internal nucleocapsid core, while the E2 and E1 transmembrane proteins on the envelope mediate receptor binding and membrane fusion. The E2 and E1 proteins associate as heterodimers during biosynthesis, and these then assemble into trimers to form spike structures on the virus surface. Alphavirus entry is initiated by the key step of virus binding to cell surface receptors (entry pathway reviewed in [12,13,14]). Virus-receptor binding is followed by clathrin-mediated uptake of the virus into the endosome compartment, where acidic pH promotes the target membrane insertion and refolding of the E1 fusion protein. This drives the fusion of the virus membrane with that of the endosome and thus delivers the nucleocapsid containing the virus genome into the host cell cytoplasm. While virus-receptor interactions promote endocytic uptake, they do not appear to play a direct role in virus-membrane fusion.

Matrix remodeling associated protein 8 (MXRA8) is a well characterized receptor for CHIKV and several other arthritogenic alphaviruses (reviewed in [12]). The ectodomain of MXRA8 is composed of 2 Ig-like domains that interact with E2-E1 heterodimers to facilitate CHIKV attachment and infection of cells in culture [15,16,17]. MXRA8 promotes CHIKV infection and pathogenesis in vivo in mice and drosophila [18]. Expression of MXRA8 is an important determinant of the infection efficiency of different cell lines. For example, there is no detectable expression of MXRA8 in Hela cells, 293T cells and A549 cells, and CHIKV infection of these cells is increased by about 1 log by exogenous MXRA8 expression [15]. Conversely, about a 1 log decrease in CHIKV infection is observed when MXRA8 is deleted from an expressing cell line such as mouse embryo fibroblasts [15]. The residual infection may reflect virus attachment to other molecules (reviewed in [12]). Together the data show that MXRA8 levels in a target cell line significantly contribute to its usefulness in CHIKV propagation and entry studies.

Baby hamster kidney (BHK) cells are one of most common vertebrate cell types used to propagate animal viruses by infection and transfection. The original BHK-21 cell cultures were established in 1961 using cells derived from the kidneys of five 1-day old Syrian golden hamsters [19]. Such kidney cell cultures are usually mixtures of fibroblasts and epithelial cells, with fibroblasts tending to predominate with time of culture [19]. A line termed clone 13 (BHK-21/C-13) was initiated by single-cell isolation [19], and is commercially available from the American Type Culture Collection. BHK-21/C-13 cells are elongated, grow in a parallel orientation, and have fibroblast-like characteristics [19]. Many viruses have been propagated in BHK-21/C-13 cells, including vaccinia virus, herpes simplex virus, vesicular stomatitis virus (VSV), and others [19]. BHK-21/C-13 cells are susceptible to alphaviruses including Sindbis virus (SINV) [20] and CHIKV [21], and have been used to produce high titer CHIKV virus stocks in suspension cultures [22].

The BHK-21/WI-2 cell line was isolated in 1965 by Vaheri et al. [23] using the technique of Macpherson and Montagnier [24], and is commercially available from Kerafast. BHK-21/WI-2 cells have a more epithelial-like morphology than BHK-21/C-13 and are reported to be efficiently transfected [25]. BHK-21/WI-2 cells have been extensively used as an alphavirus cell culture system, in particular to study Semliki Forest virus [26].

Although both of these BHK-21 cell lines have been widely employed in alphavirus research, little is known about their potential differences in alphavirus infection. Here we characterized the infectivity of SINV and CHIKV in BHK-21/C-13 and BHK-21/WI-2. While these cell lines showed comparable infection properties with SINV, we found that BHK-21/WI-2 cells were significantly less susceptible to infection by CHIKV than were BHK-21/C-13 cells. This difference was due to decreased expression of MXRA8 mRNA in BHK-21/WI-2 cells, and could be rescued by ectopic expression of MXRA8.

## 2. Materials and Methods

### 2.1. Cell Lines

BHK-21/WI-2 cells were a gift from Dr. Ari Helenius and BHK-21/C-13 cells were purchased from ATCC, CCL10. Both lines were cultured at 37 °C in high glucose Dulbecco’s modified Eagle’s medium (DMEM, HyClone, Marlborough, MA, USA) supplemented with 10% tryptose phosphate broth, 4 mM l-glutamine, 100 U penicillin/mL, and 100 μg streptomycin/mL and either 5% (BHK-21/WI-2) or 10% (BHK-21/C-13 cells) fetal bovine serum (FBS). Mouse embryonic fibroblasts (MEF) cells (a gift from Dr. Michael S. Diamond) [15] were cultured in high glucose DMEM supplemented with 10% FBS, 10 mM HEPES, 4 mM l-glutamine, 1 mM sodium pyruvate, 1× non-essential amino acids, 100 U penicillin/mL, and 100 μg streptomycin/mL.

### 2.2. Plasmids

The open reading frame (ORF) encoding the hamster MXRA8 protein (XM_005079233) plus a C-terminal FLAG tag was synthesized by Twist Bioscience (South San Francisco, CA, USA) then inserted into pCDNA3.1(−) (Invitrogen, Carlsbad, CA, USA) using the EcoRI and BamHI sites to generate the hamster-MXRA8-FLAG expression plasmid. The human-MXRA8-FLAG expression plasmid was generated by restriction digestion of a lentivirus vector (a gift from Dr. Michael S. Diamond) [15] containing the MXRA8 isoform 2 (NM_032348.3) with a C-terminal FLAG tag, and subcloned into pcDNA3.1(−) using the EcoRI and BamHI sites. Both plasmids were confirmed by sequencing.

### 2.3. Viruses

Virus stocks of CHIKV (a CHIKV/GFP 181/25 reporter virus that expresses cytoplasmic GFP during infection) (a gift from Dr. Elena I. Frolova) [27] and SINV (dsTE12Q) [28] were produced in BHK-21/WI-2 cells by electroporation of in vitro transcribed viral RNA and culture for 24 h at 37 °C. The culture media were then harvested, clarified by centrifugation, and HEPES pH 8.0 was added to a final concentration of 10 mM. Virus stocks were aliquoted and stored at −80 °C. Virus titers were measured by infectious center assays in the indicated cell lines.

### 2.4. Plaque Assays

2 × 10^5^ BHK-21/WI-2 or 3 × 10^5^ BHK-21/C-13 cells per well were seeded in 6-well plates for 24 h. CHIKV or SINV stock virus was serially diluted in Med-A (MEM plus 0.2% BSA and 10 mM HEPES pH 7.0) and added to cells for 1 h, followed by overlay with a medium containing 0.5% carboxymethylcellulose. Plaques were fixed and stained with crystal violet at 48 h post-infection.

### 2.5. Infectious Center Assay

Cells were seeded at 1 × 10^4^ cells/well in 96-well plates, cultured for ~24 h at 37 °C, infected in parallel with serially diluted CHIKV or SINV for 1.5 h at 37 °C, then cultured in media containing 20 mM NH_4_Cl at 28 °C overnight. SINV-infected cells were immunostained with mAbs R2 and R6 to SINV E1 and E2, respectively [29] CHIKV-infected cells were detected by cytosolic GFP expression. Infected cells were quantitated by fluorescence microscopy.

### 2.6. Flow Cytometry

Cells infected with CHIKV were harvested using trypsin-EDTA solution and washed two times with staining buffer (15 mM HEPES pH = 7.0 and 2% FBS in PBS). Cells were then directly fixed with 2% paraformaldehyde (PFA) for 10 min at room temperature, washed two times with staining buffer and analyzed by flow cytometry. SINV-infected cells were harvested as above and incubated with a 1:1 mixture of the mouse R6 and R2 hybridoma supernatants [29] for 1 h at 4 °C. Cells were washed twice with staining buffer and incubated with 2 μg/mL goat anti-mouse IgG (H + L) conjugated with Alexa Fluor 647 (Thermo Fisher Scientific, Waltham, MA, USA, #A21235) for 30 min at 4 °C. Cells were washed twice with staining buffer and fixed and processed as above.

For staining of MXRA8 constructs with a C-terminal FLAG tag, cells were harvested and fixed as above and permeabilized by treatment with 0.05% Triton X-100 in staining buffer for 10 min. Cells were then washed with staining buffer twice and incubated with 2.5 μg/mL anti-FLAG M2 antibody (Sigma-Aldrich, St. Louis, MO, USA, #F1804) for 1 h at 4 °C, followed by staining with goat anti-mouse IgG as above.

For all samples 1 × 10^4^ cells were analyzed by flow cytometry using a BD LSR-II analyzer (BD Biosciences, San Jose, CA, USA) in the Einstein flow cytometry core. Isotype control antibodies or mock infection were used to delineate the gates for flow analysis.

### 2.7. Electroporation Efficiency

BHK-21/WI-2 or BHK-21/C-13 cells were electroporated as previously published [30]. In brief, 4 × 10^6^ cells mixed with 7.5 μL of in vitro transcribed viral CHIKV or SINV RNA, and electroporated with two pulses of 850 V and 25 μF using a Biorad Gene Pulser II (Bio-Rad Laboratories, Hercules, CA, USA) apparatus. To measure electroporation efficiency, cells were plated in complete media for 2 h at 37 °C then cultured in media containing 20 mM NH_4_Cl at 28 °C overnight, and primary infected cells quantitated by flow cytometry. To quantitate virus spread and progeny virus production, cells were plated in complete media for 2 h then cultured in Med-A at 37 °C for 10 h. Infected cells were quantitated by flow cytometry and virus in the media was quantitated by infectious center assay in MEF cells.

### 2.8. RT-PCR and Quantitative PCR

Total cellular RNA was extracted from BHK-21/WI-2 and BHK-21/C-13 cells using Trizol according to the manufacturer’s instructions (Life Technologies, Carlsbad, CA, USA). 60 ng total RNA from each sample was used to synthesize cDNA with the Verso cDNA Synthesis Kit (Thermo Scientific, Waltham, MA, USA, #AB1453) and the included oligo(dT) primer. To confirm that these two BHK-21 cell lines are derived from the golden hamster (*Mesocricetus auratus*), the cDNAs were amplified using a primer pair targeting the golden hamster MXRA8 ORF. The sequences of the forward and reverse primers were 5′-CCGGAATTCATGGAACTGCTGTCCTGTGTC-3′ and 5′-CGCGGATCCTTATTTGCAGTACTCCTTCCTG-3′. The PCR products were purified by gel extraction and sequenced (Genewiz, South Plainfield, NJ, USA).

To compare MXRA8 RNA levels in these two BHK-21 cell lines, the cDNAs were used for qPCR using the Power SYBR Green PCR Master Mix (Thermo Scientific, #4367659) and Applied Biosystems ViiA 7 real-time PCR system. For qPCR of hamster MXRA8 the sequences of the forward and reverse primer pair #1 were 5′-CCACCACTACTGTCACCTCTA-3′ and 5′-CACCACCATGACTTCCTTCTC-3′, and those of the forward and reverse primer pair #2 were 5′-TCCCACGCTGGTAAGTAGAT-3′ and 5′-TGATGAGCAGCAAGATGAAGAG-3′. MXRA8 expression levels were normalized to those of hamster GAPDH using the forward and reverse primers 5′-GCACAGTCAAGGCTGAGAA-3′ and 5′-GCCAGTAGACTCCACAACATAC-3′.

### 2.9. Rescue Experiments

1 × 10^5^ BHK-21/WI-2 cells were seeded in 12-well plates for 24 h, then transfected with 200 ng of plasmids expressing human-MXRA8-FLAG, hamster-MXRA8-FLAG or empty vector using Lipofectamine 2000 and the manufacturer’s protocol (Thermo Scientific, #11668019). At 24 h post-transfection cells were harvested, and MXRA8-FLAG expression was analyzed by Western blot using mouse anti-FLAG M2 antibody (Sigma-Aldrich, # F1804, 1:2500), rabbit anti-human MXRA8 antibody (Sigma-Aldrich, #HPA055780, 1:1000) and mouse anti-β-actin (Sigma-Aldrich, #A5441, 1:2000) or by flow cytometry as described above. To test rescue of CHIKV infection, at 24 h post-transfection the cells were infected for 1h at 37 °C with CHIKV at an MOI = 0.5 (using a virus titer determined by infectious center assays on BHK-21/WI-2 cells). Cells were then cultured in media containing 20 mM NH_4_Cl at 28 °C overnight and analyzed by flow cytometry.

### 2.10. Statistics

As indicated in the legends, statistical significance was assessed using unpaired *t*-tests or multiple *t* tests in GraphPad Prism, version 8. The data represent the mean from at least 3 independent experiments.

## 3. Results

### 3.1. BHK-21/WI-2 Cells Are Less Susceptible to CHIKV Than Are BHK-21/C-13 Cells

**Figure 1:**
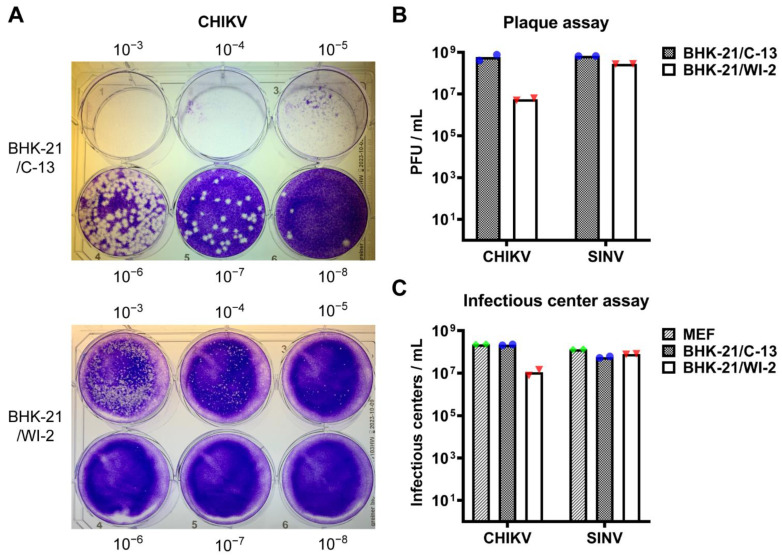
Infectivity of CHIKV in BHK-21/C-13 and BHK-21/WI-2 cells. (**A**,**B**) BHK-21/C-13 cells or BHK-21/WI-2 cells were infected in parallel with serial dilutions of CHIKV or SINV and overlaid with medium containing 0.5% CMC. Plaques were fixed and stained at 48 h post-infection. Representative images from one of 2 independent experiments are shown in (**A**). The bar graph in (**B**) shows the mean and individual data points of 2 independent experiments. (**C**) Infectious center assay. MEF, BHK-21/C-13, or BHK-21/WI-2 cells were infected in parallel with serial dilutions of CHIKV or SINV. Primary infection was quantitated by immunofluorescence. Data shown are the mean and individual data points of 2 independent experiments.

We compared the infectivity of CHIKV in these two BHK-21 cell clones by performing plaque assays in parallel using the same virus stock. The plaque size in BHK-21/WI-2 cells was considerably smaller than that in BHK-21/C-13 cells (Figure 1A). The titer of the CHIKV stock obtained by plaque assays on BHK-21/C-13 cells was 5.8 × 10^8^ PFU/mL while the titer obtained by plaque assays on BHK-21/WI-2 was 5.6 × 10^6^ PFU/mL, approximately 2-logs lower. By contrast, minimal difference in infection or plaque size was observed for SINV between the two BHK-21 cell clones (Figure 1B and data not shown). To avoid any effects of the reduced plaque size in quantitating CHIKV infection, we then used an infectious center assay to compare CHIKV infectivity in the two BHK-21 cell clones vs. in mouse embryo fibroblasts (MEF) cells, which express the MXRA8 receptor and are highly susceptible to CHIKV [15]. CHIKV infectivity in BHK-21/C-13 cells was comparable to that in MEF cells while infectivity was decreased by more than 1-log in BHK-21/WI-2 cells (Figure 1C).

**Figure 2:**
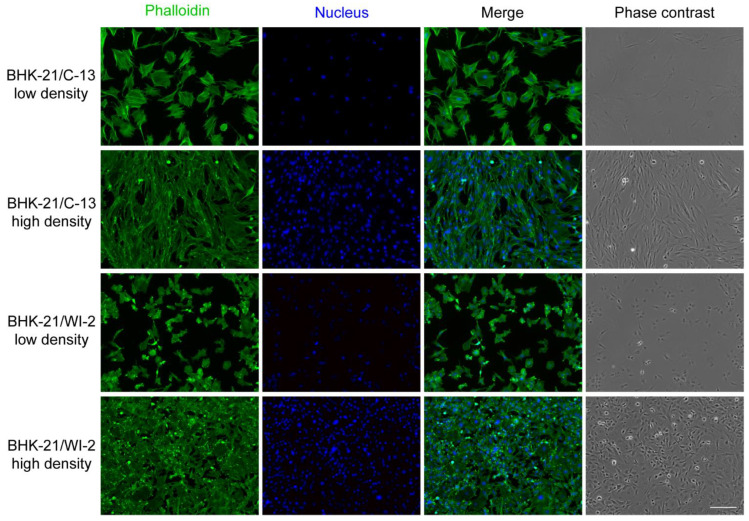
Morphology of BHK-21/C-13 and BHK-21/WI-2 cells. BHK-21/C-13 or BHK-21/WI-2 cells were seeded at low or high density, cultured for 24 h, and stained for actin with phalloidin (green) and for DNA with Hoechst (blue). Cells were imaged by fluorescence microscopy. Scale bar = 150 µm.

In agreement with previous reports, we observed morphological differences between these two BHK-21 cell lines in culture (Figure 2). BHK-21/C-13 were fibroblast-like cells and had elongated shapes. BHK-21/WI-2 were epithelial-like cells and more polygonal in shape. These distinct morphologies made the two cell lines readily distinguishable in culture (Figure 2). To confirm that both of these BHK-21 cell clones are derived from the golden hamster, we isolated RNA from both lines and used RT-PCR to amplify the open reading frame of the MXRA8 receptor cDNA. Sequence analysis indicated that both cell lines contained mRNA identical to transcript variant X1 of golden hamster MXRA8 (accession: XM_005079233) (data not shown).

### 3.2. Electroporation Efficiency of Alphavirus RNA into BHK-21/WI-2 and BHK-21/C-13 Cells

**Figure 3:**
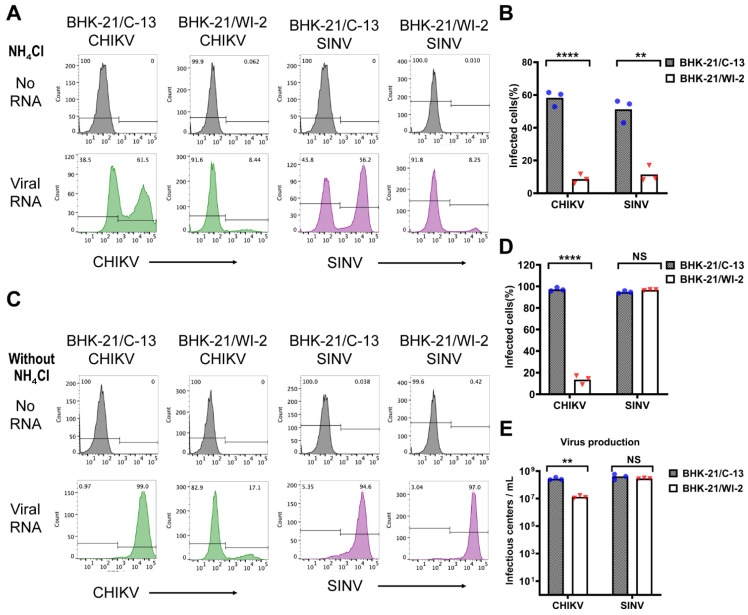
Viral RNA electroporation efficiency of BHK-21/C-13 and BHK-21/WI-2 cells. Equivalent numbers of BHK-21/C-13 or BHK-21/WI-2 cells were electroporated in parallel with in vitro transcribed CHIKV or SINV viral RNA. (**A**,**B**) After electroporation, cells were cultured in complete media for 2 h at 37 °C, then incubated at 28 °C overnight in media containing 20 mM NH_4_Cl to prevent secondary infection. (**A**) Infection was quantitated by flow cytometry. A representative example of 3 independent experiments is shown. (**B**) The bar graph represents the mean and individual data points of the % infection from 3 independent experiments. (**C**–**E**) After electroporation, cells were cultured in complete media for 2 h, then cultured in Med-A in the absence of NH_4_Cl for 10 h. (**C**) Infection was quantitated by flow cytometry. A representative example of 3 independent experiments is shown. (**D**) The bar graph represents the mean and individual data points of the % infection from 3 independent experiments. (**E**) The virus released in the medium was quantitated by infectious center assay. The bar graph shows the mean and individual data points from 3 independent experiments. For panels (**B**,**D**,**E**), multiple *t* test was performed to assess the statistical significance of differences between BHK-21/C-13 and BHK-21/WI-2 cells. **, *p* < 0.01; ****, *p* < 0.0001.

Since BHK-21 cells are widely used to generate alphavirus stocks by electroporation of in vitro transcribed viral RNAs, we analyzed whether electroporation efficiency was comparable between these two BHK-21 cell clones. BHK-21/WI-2 and BHK-21/C-13 were electroporated in parallel with in vitro transcribed CHIKV or SINV RNA. The cells were then cultured in the presence of NH_4_Cl to prevent secondary infection and analyzed by flow cytometry. The results showed that the electroporation efficiency of either CHIKV or SINV RNA in BHK-21/WI-2 was significantly lower than that of BHK-21/C-13 (Figure 3A,B). To measure virus spread and progeny virus production in these BHK-21 cell lines, after electroporation the cells were cultured in the absence of NH_4_Cl for 12 h. Infected cells were then analyzed by flow cytometry and the virus in the cell media was quantitated by infectious center assay. Under these conditions most of BHK-21/C-13 cells became infected by CHIKV (average 97.2% in 3 experiments), while spread in the BHK-21/WI-2 cells was significantly reduced (average 13.5% in 3 experiments) (Figure 3C,D). By contrast, comparable spread of SINV infection was observed in these two BHK-21 cell clones (Figure 3C,D). CHIKV production was also significantly reduced in BHK-21/WI-2 cells vs BHK-21/C-13, while the production of SINV was similar between the two cell clones (Figure 3E). Thus, while the electroporation efficiency of either alphavirus RNA was lower in BHK-21/WI-2 than in BHK-21/C-13, CHIKV spread and virus production was specifically reduced in BHK-21/WI-2.

### 3.3. Expression of MXRA8 RNA in BHK-21/WI-2 and BHK-21/C-13 Cells

**Figure 4:**
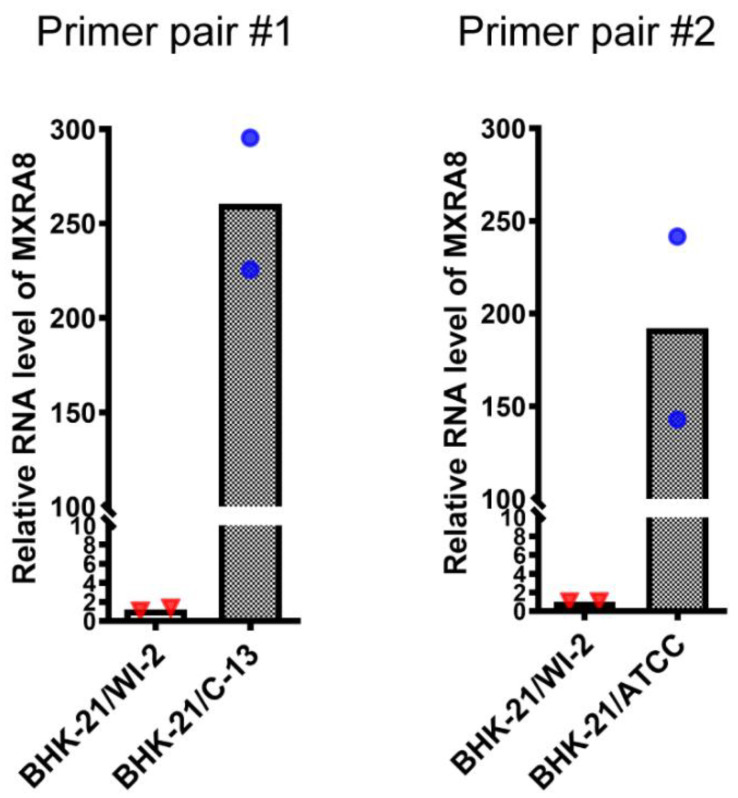
Quantitation of MXRA8 RNA in BHK-21/WI-2 and BHK-21/C-13 cells. Total RNA in BHK-21/WI-2 and BHK-21/C-13 cells was extracted and reverse-transcribed. The level of hamster MXRA8 RNA was measured by qPCR using two different primer pairs as described in the methods. Data shown are the mean and individual data points of 2 independent experiments, with the level of MXRA8 RNA in the BHK-21/WI-2 cells set to 1.

We hypothesized that the expression of the CHIKV receptor MXRA8 could be responsible for the differences in CHIKV infection between these two BHK-21 cell clones. Since there is no available commercial antibody against the hamster MXRA8, we tested a monoclonal antibody (mAb) against mouse MXRA8 (9G2.D6) [15] and a commercial mAb against human MXRA8 for their ability to recognize hamster MXRA8. Both antibodies failed to detect hamster MXRA8 in sensitive flow cytometry experiments (data not shown). We therefore measured the mRNA levels of hamster MXRA8 in these two BHK-21 cell clones by quantitative PCR analysis using two different primer pairs targeting hamster MXRA8. As shown in Figure 4, using either primer pair the level of MXRA8 mRNA in BHK-21/WI-2 was more than 150-times lower than that in BHK-21/C-13.

### 3.4. Exogenous Expression of Either Human MXRA8 or Hamster MXRA8 Rescues CHIKV Infection of BHK-21/WI-2 Cells

**Figure 5:**
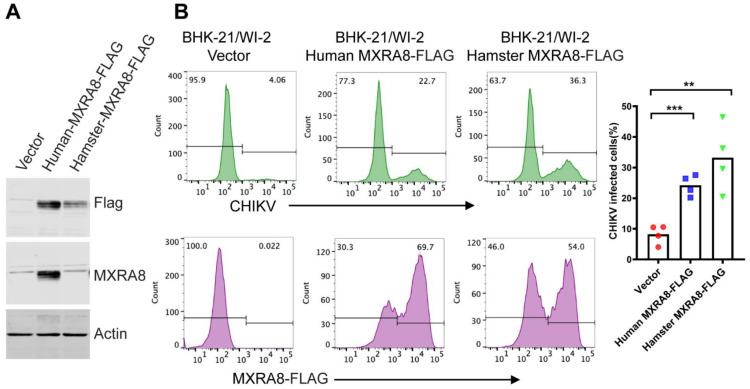
CHIKV infection of BHK-21/WI-2 cells is rescued by expression of human or hamster MXRA8. (**A**) Protein expression by human or hamster MXRA8 plasmids. BHK-21/WI-2 cells were transfected with constructs encoding human-MXRA8-Flag or hamster-MXRA8-Flag, or with empty vector (pcDNA3.1), and cultured for 24 h. Cell lysates were analyzed by Western blot with an antibody to the Flag tag or with an antibody that specifically recognizes human MXRA8. (**B**) CHIKV infection in BHK-21/WI-2 cells expressing human or hamster MXRA8. BHK-21/WI-2 cells were transfected with human-MXRA8-Flag, hamster-MXRA8-Flag or empty vector and cultured for 24 h. Cells were then infected with CHIKV. After a 1 h infection the cells were incubated overnight in medium containing 20 mM NH_4_Cl to prevent secondary infection, and MXRA8 expression and CHIKV infection were quantitated by flow cytometry. (**B**) The flow data on the left are a representative example of 4 independent experiments. The bar graph on the right shows the mean and individual data points of the % infection from 4 independent experiments. Unpaired *t* test was performed to assess the statistical significance of differences in CHIKV infection in cells expressing human/hamster MXRA8 compared to that in vector control. **, *p* < 0.01; ***, *p* < 0.001. The difference between infection of cells expressing human vs. hamster MXRA8 is not statistically significant (*p* = 0.167).

To determine the role of low MXRA8 expression in reducing CHIKV infection, we expressed FLAG-tagged human MXRA8 or hamster MXRA8 by transient transfection of BHK-21/WI-2 cells. Following transfection, expression of both proteins was detected by Western blot using a mAb to the FLAG tag, while only the human MXRA8 was detected by a polyclonal antibody against human MXRA8 (Figure 5A). Expression of either human or hamster MXRA8 resulted in a significant increase in CHIKV infection of BHK-21/WI-2 (Figure 5B), thus confirming that the reduced infection was caused by reduced MXRA8 expression.

## 4. Discussion

Here we compared CHIKV infectivity in two BHK-21 clonal cell lines, both of which were isolated from the original BHK-21 cell culture [19,23] and both of which are widely used in alphavirus research. The morphology of these two BHK-21 cell lines was distinct, with BHK-21/WI-2 cells being more epithelial-like and BHK-21/C-13 being more fibroblast-like. Both cell lines were equivalently infected by SINV. Primary CHIKV infection in BHK-21/WI-2 cells was significantly lower than that in BHK-21/C-13, and BHK-21/WI-2 cells also produced smaller plaques and lower progeny virus titers. The reduced CHIKV infectivity in the BHK-21/WI-2 cell line was shown to be due to its relatively low expression of the CHIKV receptor MXRA8. Important species differences have been observed in the receptor competency of MXRA8. While MXRA8 from various mammals including mouse, human, rat, and dog support CHIKV infection, cattle MXRA8 is inactive [31]. The structurally identified contact sites of CHIKV with human MXRA8 indicate six differences vs. the sequence of the hamster receptor [17]. Structural and mutagenesis studies of mouse MXRA8 defined 5 residues that were influential in CHIKV binding [16], and all of these are conserved among the human, mouse, and hamster receptors. Our results show that CHIKV infection of BHK-21/WI-2 cells was rescued by ectopic expression of either human or hamster MXRA8, thus confirming that hamster MXRA8 is an active CHIKV receptor.

BHK-21 cells are the basis of a well-established and widely used electroporation protocol to deliver in vitro transcribed viral RNAs and generate virus stocks [30,32]. Using either SINV or CHIKV RNA, we found that the electroporation efficiency of BHK-21/WI-2 cells was lower than that of BHK-21/C-13. Despite this, comparable final titers of SINV were produced from the two cell lines, since SINV secondary infection was efficient in both. Conversely, the reduced secondary infection of CHIKV in BHK-21/WI-2 cells led to a strong reduction in the final virus titer. It is possible that alterations in the established electroporation protocol could produce a higher efficiency of alphavirus RNA delivery into BHK-21/WI-2 cells. Under the published conditions, generation of CHIKV stocks from infectious clone-derived RNAs is more productive in BHK-21/C-13 cells.

Unlike RNA electroporation, transfection of DNA plasmids was more efficient in BHK-21/WI-2 cells than in BHK-21/C-13 cells. We found that the transfection efficiency of the human MXRA8 expression plasmid was ~54% in BHK-21/WI-2 vs. ~31% in BHK-21/C-13 (*n* = 4). These efficient transfection properties have made BHK-21/WI-2 cells a preferred system to recover infectious recombinant VSV [33,34], and for the recovery and amplification of replication-restricted ΔG-VSV [25]. For example, BHK-21/WI-2 cells were recently used to generate VSV pseudoviruses bearing the SARS-CoV-2 S protein to evaluate neutralizing antibodies [35]. Thus, each of these BHK cell lines has unique advantages depending on the experimental system.

Transmission of CHIKV to humans occurs via the bite of an infected mosquito and leads to initial infection of cells at the site. The virus subsequently spreads via the bloodstream, infecting a variety of tissues especially musculoskeletal tissues [3]. In humans, macrophages, fibroblasts, endothelial and epithelial cells are susceptible to infection. Mouse models and human biopsies have identified fibroblasts as the main CHIKV target in peripheral tissues [3,36]. In keeping with this in vivo tropism, we found that the fibroblast-like BHK-21/C-13 cells were more susceptible to CHIKV infection than were the epithelial-like BHK-21/WI-2 cells. The Human Protein Atlas database reports robust expression of MXRA8 mRNA in human fibroblasts and lower levels of expression in cells identified as epithelial, suggesting that epithelial cells in vivo may have a similar phenotype as BHK-21/WI-2 cells [37].

While a number of studies have reported the susceptibility of BHK-21 cells to CHIKV infection, the identity of the BHK-21 cell clone is not always specified [38,39], [but see also 21]. In addition, other BHK-21 cell lines have been derived from the original BHK-21 cultures and some, such as BHK-21/15, are useful for alphavirus studies (e.g., [40]). To our knowledge the relative MXRA8 receptor levels of other BHK-21 cell clones have not been characterized. MXRA8 also acts as a receptor for infection by the alphaviruses Mayaro, Ross River and O’nyong nyong virus [15]. The infectivity of these arthritogenic alphaviruses in BHK-21/WI-2 could also be impacted by the low expression of MXRA8. Together our results highlight the importance of consideration of the properties and receptor expression level when selecting a host cell line for virus studies.

## References

[1] Powers A.M., Brault A.C., Shirako Y., Strauss E.G., Kang W., Strauss J.H., Weaver S.C. (2001). Evolutionary relationships and systematics of the alphaviruses. J. Virol..

[2] Kuhn R.J., Howley P.M., Knipe D.M. (2021). Togaviridae: The Viruses and Their Replication. Fields Virology: Emerging Viruses.

[3] Silva L.A., Dermody T.S. (2017). Chikungunya virus: Epidemiology, replication, disease mechanisms, and prospective intervention strategies. J. Clin. Investig..

[4] Mason P.J., Haddow A.J. (1957). An epidemic of virus disease in Southern Province, Tanganyika Territory, in 1952-53; an additional note on Chikungunya virus isolations and serum antibodies. Trans. R. Soc. Trop. Med. Hyg..

[5] Weaver S.C., Winegar R., Manger I.D., Forrester N.L. (2012). Alphaviruses: Population genetics and determinants of emergence. Antivir. Res..

[6] Enserink M. (2007). Infectious diseases. Chikungunya: No longer a third world disease. Science.

[7] Schwartz O., Albert M.L. (2010). Biology and pathogenesis of chikungunya virus. Nat. Rev. Microbiol..

[8] Vazeille M., Moutailler S., Coudrier D., Rousseaux C., Khun H., Huerre M., Thiria J., Dehecq J.S., Fontenille D., Schuffenecker I. (2007). Two Chikungunya Isolates from the Outbreak of La Reunion (Indian Ocean) Exhibit Different Patterns of Infection in the Mosquito, Aedes albopictus. PLoS ONE.

[9] Johansson M.A. (2015). Chikungunya on the move. Trends Parasitol..

[10] Morrison T.E. (2014). Reemergence of chikungunya virus. J. Virol..

[11] Brown R.S., Wan J.J., Kielian M. (2018). The Alphavirus Exit Pathway: What We Know and What We Wish We Knew. Viruses.

[12] Holmes A.C., Basore K., Fremont D.H., Diamond M.S. (2020). A molecular understanding of alphavirus entry. PLoS Pathog..

[13] Kielian M. (2014). Mechanisms of Virus Membrane Fusion Proteins. Annu. Rev. Virol..

[14] Kielian M., Chanel-Vos C., Liao M. (2010). Alphavirus entry and membrane fusion. Viruses.

[15] Zhang R., Kim A.S., Fox J.M., Nair S., Basore K., Klimstra W.B., Rimkunas R., Fong R.H., Lin H., Poddar S. (2018). Mxra8 is a receptor for multiple arthritogenic alphaviruses. Nature.

[16] Basore K., Kim A.S., Nelson C.A., Zhang R., Smith B.K., Uranga C., Vang L., Cheng M., Gross M.L., Smith J. (2019). Cryo-EM Structure of Chikungunya Virus in Complex with the Mxra8 Receptor. Cell.

[17] Song H., Zhao Z., Chai Y., Jin X., Li C., Yuan F., Liu S., Gao Z., Wang H., Song J. (2019). Molecular Basis of Arthritogenic Alphavirus Receptor MXRA8 Binding to Chikungunya Virus Envelope Protein. Cell.

[18] Zhang R., Earnest J.T., Kim A.S., Winkler E.S., Desai P., Adams L.J., Hu G., Bullock C., Gold B., Cherry S. (2019). Expression of the Mxra8 Receptor Promotes Alphavirus Infection and Pathogenesis in Mice and Drosophila. Cell Rep..

[19] Stoker M., Macpherson I. (1964). Syrian Hamster Fibroblast Cell Line Bhk21 and Its Derivatives. Nature.

[20] Eaton B.T., Faulkner P. (1973). Altered pattern of viral RNA synthesis in cells infected with standard and defective Sindbis virus. Virology.

[21] Lee C.H.R., Mohamed Hussain K., Chu J.J.H. (2019). Macropinocytosis dependent entry of Chikungunya virus into human muscle cells. PLoS Negl. Trop. Dis..

[22] Davis J.L., Hodge H.M., Campbell W.E. (1971). Growth of chikungunya virus in baby hamster kidney cell (BHK-21-clone 13) suspension cultures. Appl. Microbiol..

[23] Vaheri A., Sedwick W.D., Plotkin S.A., Maes R. (1965). Cytopathic effect of rubella virus in RHK21 cells and growth to high titers in suspension culture. Virology.

[24] Macpherson I., Stoker M. (1962). Polyoma transformation of hamster cell clones--an investigation of genetic factors affecting cell competence. Virology.

[25] Whitt M.A. (2010). Generation of VSV pseudotypes using recombinant DeltaG-VSV for studies on virus entry, identification of entry inhibitors, and immune responses to vaccines. J. Virol. Methods.

[26] Helenius A., Soderlund H. (1973). Stepwise dissociation of the Semliki Forest Virus membrane with triton X-100. Biochim. Biophys. Acta.

[27] Reynaud J.M., Kim D.Y., Atasheva S., Rasalouskaya A., White J.P., Diamond M.S., Weaver S.C., Frolova E.I., Frolov I. (2015). IFIT1 Differentially Interferes with Translation and Replication of Alphavirus Genomes and Promotes Induction of Type I Interferon. PLoS Pathog..

[28] Hardwick J.M., Levine B. (2000). Sindbis virus vector system for functional analysis of apoptosis regulators. Meth. Enzym..

[29] Meyer W.J., Gidwitz S., Ayers V.K., Schoepp R.J., Johnston R.E. (1992). Conformational alteration of Sindbis virion glycoproteins induced by heat, reducing agents, or low pH. J. Virol..

[30] Liljestrom P., Lusa S., Huylebroeck D., Garoff H. (1991). In vitro mutagenesis of a full-length cDNA clone of Semliki Forest virus: The small 6,000-molecular-weight membrane protein modulates virus release. J. Virol..

[31] Kim A.S., Zimmerman O., Fox J.M., Nelson C.A., Basore K., Zhang R., Durnell L., Desai C., Bullock C., Deem S.L. (2020). An Evolutionary Insertion in the Mxra8 Receptor-Binding Site Confers Resistance to Alphavirus Infection and Pathogenesis. Cell Host Microbe.

[32] Liljestrom P., Garoff H. (1991). A new generation of animal cell expression vectors based on the Semliki Forest virus replicon. Biotechnology.

[33] Lawson N.D., Stillman E.A., Whitt M.A., Rose J.K. (1995). Recombinant vesicular stomatitis viruses from DNA. Proc. Natl. Acad. Sci. USA.

[34] Jayakar H.R., Murti K.G., Whitt M.A. (2000). Mutations in the PPPY motif of vesicular stomatitis virus matrix protein reduce virus budding by inhibiting a late step in virion release. J. Virol..

[35] Hashem A.M., Algaissi A., Almahboub S.A., Alfaleh M.A., Abujamel T.S., Alamri S.S., Alluhaybi K.A., Hobani H.I., AlHarbi R.H., Alsulaiman R.M. (2020). Early Humoral Response Correlates with Disease Severity and Outcomes in COVID-19 Patients. Viruses.

[36] Couderc T., Lecuit M. (2009). Focus on Chikungunya pathophysiology in human and animal models. Microbes Infect..

[37] Uhlen M., Fagerberg L., Hallstrom B.M., Lindskog C., Oksvold P., Mardinoglu A., Sivertsson A., Kampf C., Sjostedt E., Asplund A. (2015). Proteomics. Tissue-based map of the human proteome. Science.

[38] Sudeep A.B., Vyas P.B., Parashar D., Shil P. (2019). Differential susceptibility & replication potential of Vero E6, BHK-21, RD, A-549, C6/36 cells & Aedes aegypti mosquitoes to three strains of chikungunya virus. Indian J. Med. Res..

[39] Roberts G.C., Zothner C., Remenyi R., Merits A., Stonehouse N.J., Harris M. (2017). Evaluation of a range of mammalian and mosquito cell lines for use in Chikungunya virus research. Sci. Rep..

[40] Pal P., Dowd K.A., Brien J.D., Edeling M.A., Gorlatov S., Johnson S., Lee I., Akahata W., Nabel G.J., Richter M.K. (2013). Development of a highly protective combination monoclonal antibody therapy against Chikungunya virus. PLoS Pathog..

